# Cr(VI) Adsorption by Mg/Al Layered Double Hydroxide-Modified Sphagnum Moss Cellulose Gel: Performance and Mechanism

**DOI:** 10.3390/molecules30081796

**Published:** 2025-04-17

**Authors:** Junpeng Ren, Shijiang Zhang, Yu Wang, Huixian Shi, Cheng Zhen

**Affiliations:** School of Chemistry and Materials Science, Guizhou Education University, Guiyang 550018, China; rjunpeng@126.com (J.R.); 18212717435@163.com (S.Z.); fengqingluanshu@126.com (H.S.); chemdoctor@foxmai.com (C.Z.)

**Keywords:** sphagnum moss cellulose gel, surface modification, layered double hydroxide (LDH), Cr(VI) removal, adsorption mechanism

## Abstract

Hexavalent chromium (Cr(VI)), a highly toxic and carcinogenic contaminant, presents a significant hazard to aquatic ecosystems and human health. Developing environmentally friendly, cost-effective, biodegradable, and easily recyclable adsorbents is critical for efficient Cr(VI) removal. Here, we present an innovative solution using a Mg/Al layered double hydroxide (LDH)-modified sphagnum cellulose gel (MgAl/LDH@SMCG), prepared by pre-treating sphagnum cellulose, crosslinking with polyvinyl alcohol, and doping with LDH. The resulting porous composite gel features abundant -COOH and -OH chelating groups, significantly enhancing its adsorption capacity and structural stability. The material’s structure and surface modifications were systematically characterized using SEM, TGA, FT-IR, and XPS. Batch adsorption experiments were conducted to assess the influence of adsorbent dosage, initial Cr(VI) concentration, pH, contact time, and temperature on performance. Adsorption kinetics, isotherms, and thermodynamics analyses revealed a primary mechanism of monolayer chemical adsorption, with experimental data closely fitting the Freundlich isotherm and pseudo-second-order kinetic models. The modified gel exhibits increased surface roughness and adsorption sites, resulting in markedly improved Cr(VI) removal efficiency. This study not only provides theoretical insights into Cr(VI) adsorption but also highlights the potential of LDH-functionalized cellulose gels for heavy metal wastewater treatment, offering a sustainable pathway for addressing global water contamination challenges.

## 1. Introduction

Environmental pollution, especially water contamination, has become a significant global challenge. Water quality is closely linked to human health; however, rapid industrialization frequently results in the excessive discharge of toxic pollutants. The release of industrial and biological wastewater has significantly altered the composition of aquatic ecosystems, further aggravating pollution concerns [1,2]. Heavy metal contamination is a critical aspect of water pollution, with hexavalent chromium (Cr(VI)) attracting significant attention due to its extreme toxicity, carcinogenicity, and mutagenicity [3,4]. Cr(VI) is widely present in industrial wastewater originating from leather tanning, electroplating, painting, paper production, and battery manufacturing. Owing to its high mobility and bioaccumulative nature, Cr(VI) poses significant threats to ecosystems and human health, potentially causing lung cancer, skin irritation, and respiratory disorders [5,6,7]. Thus, the development of efficient Cr(VI) removal technologies is essential for protecting aquatic environments and public health [3].

Various technologies for Cr(VI) removal from wastewater include ion exchange, chemical precipitation, photocatalysis, and bioadsorption [8,9,10]. However, many of these methods, including chemical precipitation and photocatalysis, are limited in practical applications due to high costs, process complexity, and secondary pollution risks [11]. In contrast, adsorption is regarded as one of the most promising methods for Cr(VI) removal due to its cost-effectiveness, high efficiency, eco-friendliness, and operational simplicity [12,13]. The design and synthesis of high-performance adsorbents are vital for achieving efficient Cr(VI) removal. Effective adsorbents typically require accessibility, functionalized surfaces, well-developed porosity, and high specific surface area [14]. Adsorbent materials are generally classified into two categories: inorganic adsorbents and polymer-based adsorbents. Common inorganic adsorbents, including activated carbon, zeolites, and kaolinite, possess excellent thermal and chemical stability. However, their limited adsorption sites, relatively low surface area, and poor reusability often restrict their effectiveness in treating high-concentration or large-volume wastewater [15]. In contrast, polymer-based adsorbents have attracted growing interest in recent years due to their ease of synthesis, high adsorption efficiency, facile surface functionalization, tunable structures, and eco-friendliness [11,16]. Notably, gel-based materials possess unique three-dimensional polymer networks and abundant functional groups, facilitating strong interactions with heavy metal ions in solution, thereby enhancing adsorption efficiency [12]. Among them, cellulose-based gels, derived from natural and renewable sources, possess abundant hydroxyl groups, well-developed porous structures, and high specific surface areas, making them inherently advantageous for the adsorption of metal ions and organic pollutants [17]. However, the dense molecular arrangement of cellulose-based gels often hinders the exposure of active adsorption sites [18]. Additionally, their susceptibility to contamination and aging during prolonged use compromises adsorption efficiency and cycling stability. Consequently, enhancing the surface properties and structural characteristics of cellulose gels to improve their adsorption performance has emerged as a critical research focus.

Layered double hydroxides (LDHs) are a class of inorganic materials characterized by a unique layered structure, comprising positively charged metal hydroxide layers and interlayer anions that are exchangeable [19]. Owing to their facile synthesis, high specific surface area, high layer charge density, and superior ion exchange capacity, LDHs have recently gained significant attention in water treatment applications [20]. Mg/Al LDH, a representative member of this class, demonstrates outstanding performance in removing heavy metal ions from wastewater [21,22]. The abundant active sites in the interlayer region strongly complex with Cr(VI), thereby enhancing adsorption efficiency. However, LDH materials frequently experience reduced specific surface area and partial shielding of active sites due to their intrinsic two-dimensional stacked structure, thereby limiting their contact efficiency with pollutants. Additionally, standalone LDH materials exhibit poor mechanical stability and tend to aggregate upon repeated use, which compromises their stability and reusability [23]. Thus, immobilizing LDHs onto a high-surface-area, porous substrate not only mitigates their inherent limitations but also harnesses the benefits of the supporting matrix, making it a promising strategy for improving Cr(VI) removal efficiency.

Sphagnum moss is a widely available and renewable plant commonly found in humid regions such as Brazil, Ireland, China, and the United Kingdom. It is primarily composed of cellulose and lignin, and features a naturally porous structure, high specific surface area, and excellent biodegradability [24,25]. These properties, combined with its non-toxicity, low cost, and environmental compatibility, make sphagnum moss an attractive candidate for environmental applications. In particular, sphagnum moss contains abundant oxygen-containing functional groups and aromatic moieties that provide numerous active sites for the adsorption of both organic and inorganic pollutants. Moreover, it possesses a high cation exchange capacity, enabling the efficient removal of heavy metals and dyes through ion exchange and complexation [26]. Compared with other cellulose sources, sphagnum moss offers advantages such as ecological adaptability, unique structural features, and superior water-holding capacity. Although its application as a natural adsorbent has been extensively studied, the use of sphagnum moss in gel-based material development remains relatively unexplored. To address this gap, we designed a novel composite adsorbent—MgAl/LDH-modified sphagnum moss cellulose gel (MgAl/LDH@SMCG). In this study, sphagnum moss cellulose was pretreated and subsequently crosslinked with polyvinyl alcohol (PVA) to construct a cellulose-based gel featuring a stable three-dimensional porous network suitable for water treatment applications. Subsequently, Mg/Al LDH was integrated into the cellulose gel matrix via an optimized loading method, forming a stable organic–inorganic composite. The composite retains the high porosity, biodegradability, eco-friendliness, and cost-effectiveness of cellulose gels while incorporating the abundant adsorption sites and superior ion exchange capacity of LDHs. This synergistic integration markedly enhances the adsorption rate and capacity for Cr(VI), while improving structural stability and reusability. Systematic characterization and adsorption experiments elucidate the adsorption mechanism of MgAl/LDH@SMCG and confirm its high efficiency in Cr(VI) removal. The design and mechanistic study of MgAl/LDH@SMCG provide a novel technological approach for efficient Cr(VI) removal. Furthermore, the organic–inorganic composite strategy enables simultaneous optimization of material structure and functionality, overcoming the inherent limitations of conventional cellulose gels in adsorption efficiency and stability. Additionally, this study explores the potential of sphagnum moss cellulose, a relatively underutilized natural resource, in expanding the polymeric adsorbent material system. This work provides new research insights and potential practical applications in wastewater treatment.

## 2. Results and Discussion

### 2.1. Analyses of Morphologies

**Figure 1:**
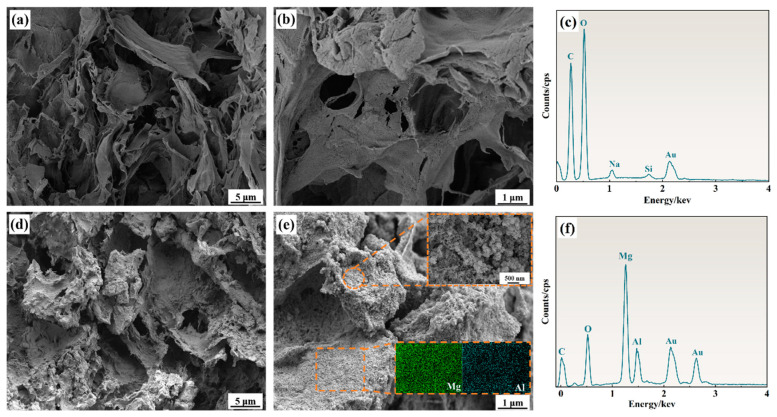
(**a**,**b**) SEM images of pristine SMCG and (**d**,**e**) SEM images of MgAl/LDH@SMCG, with corresponding elemental mappings of Mg and Al; (**c**,**f**) EDS spectra of SMCG and MgAl/LDH@SMCG.

The surface morphology of the sphagnum moss gel (SMCG) adsorbent, both before and after loading, was analyzed using scanning electron microscopy (SEM) (Figure 1). The original sphagnum moss gel (SMCG) (Figure 1a,b) exhibited a smooth, flat surface with a well-organized porous structure. This structure features a complex interwoven network of cavities, providing ideal channels for the rapid transfer and diffusion of heavy metal ions, demonstrating its potential as an efficient adsorbent. After loading with Mg-Al/LDH, the sphagnum moss gel adsorbent maintained its porous and fibrous structure. Numerous fine particles were uniformly attached to its surface, significantly increasing surface roughness. Magnified images reveal that the uniformly loaded Mg-Al LDH exhibit characteristic hydrotalcite-like layered structures with hexagonal platelet morphologies [27]. Compared to the original smooth surface of the sphagnum moss gel, the MgAl/LDH@SMCG composite exhibited an obviously rougher and more textured surface morphology after LDH loading, as observed in SEM images. This increased surface heterogeneity is likely to facilitate Cr(VI) adsorption by providing more contact points and enhancing surface interaction.

Additionally, EDS spectrum analysis confirmed the presence of Mg and Al elements in the MgAl/LDH@SMCG adsorbent after loading (Figure 1c,f), indicating changes in surface composition. To assess the uniformity of surface loading, the distribution of Mg and Al elements on the loaded MgAl/LDH@SMCG surface was characterized (Figure 1e). The results showed that Mg-Al LDHs were uniformly distributed across the entire SMCG surface, indicating a strong affinity between LDH and SMCG. This uniform distribution significantly enhanced the stability and adsorption performance of the adsorbent.

### 2.2. FT-IR Analysis

**Figure 2:**
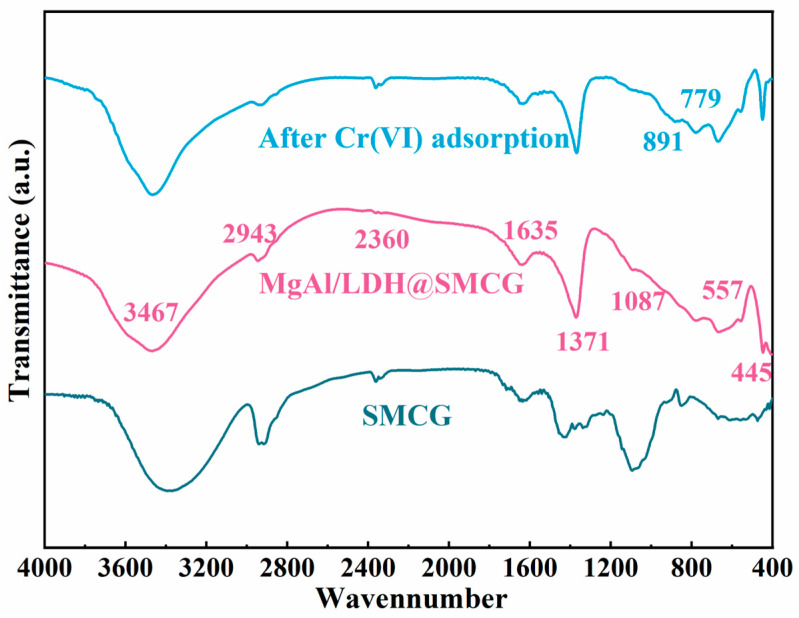
FT−IR spectra of SMCG, MgAl/LDH@SMCG, and Cr(VI)−adsorbed MgAl/LDH@SMCG.

To investigate the changes in surface structure resulting from modification and Cr(VI) adsorption, FT-IR analysis was performed on SMCG, MgAl-LDH@SMCG, and Cr(VI)-adsorbed MgAl-LDH@SMCG samples (Figure 2). The results indicate that the absorption peak at 3467 cm^−1^ in SMCG, both before and after surface modification, corresponds to the O–H stretching vibration of surface or interlayer water. Furthermore, the absorption peak at 1635 cm^−1^ is ascribed to the bending vibration of physically adsorbed water on the adsorbent surface, which facilitates hydrogen-bonding interactions during adsorption [4,28]. All three samples exhibit a prominent absorption peak at 2943 cm^−1^, attributed to the stretching vibration of C–H bonds [29]. However, following modification and Cr(VI) adsorption, the intensity of this peak significantly decreases, suggesting that the C–H bonds were disrupted during the modification process. The absorption peak at 1087 cm^−1^ is assigned to the stretching vibration of the C–O bond [1,30], while the peak at 2360 cm^−1^ originates from the stretching vibration of carbon–carbon double bonds (C=O) [31]. Furthermore, the absorption peak at 1371 cm^−1^ likely corresponds to the vibration of carboxylate ligands [27], which serve as active sites for complexation during adsorption. Compared with the original SMCG, the MgAl-LDH@SMCG sample exhibits two new absorption peaks in the low-frequency region at 557 cm^−1^ and 445 cm^−1^. These peaks are attributed to a layered structure formed by metal–hydroxide and metal–hydroxide–metal bonds (e.g., Mg–O and Al–O) [32,33], confirming the successful loading of Mg–Al LDHs onto the sample. After Cr(VI) adsorption, the MgAl-LDH@SMCG sample exhibits two new absorption peaks at 779 cm^−1^ and 891 cm^−1^, attributed to the vibration of Cr–O and Cr=O bonds [34,35]. This observation indicates that HCrO_4_^−^ and CrO_4_^2−^ have been successfully adsorbed onto the material.

### 2.3. XPS Analysis

**Figure 3:**
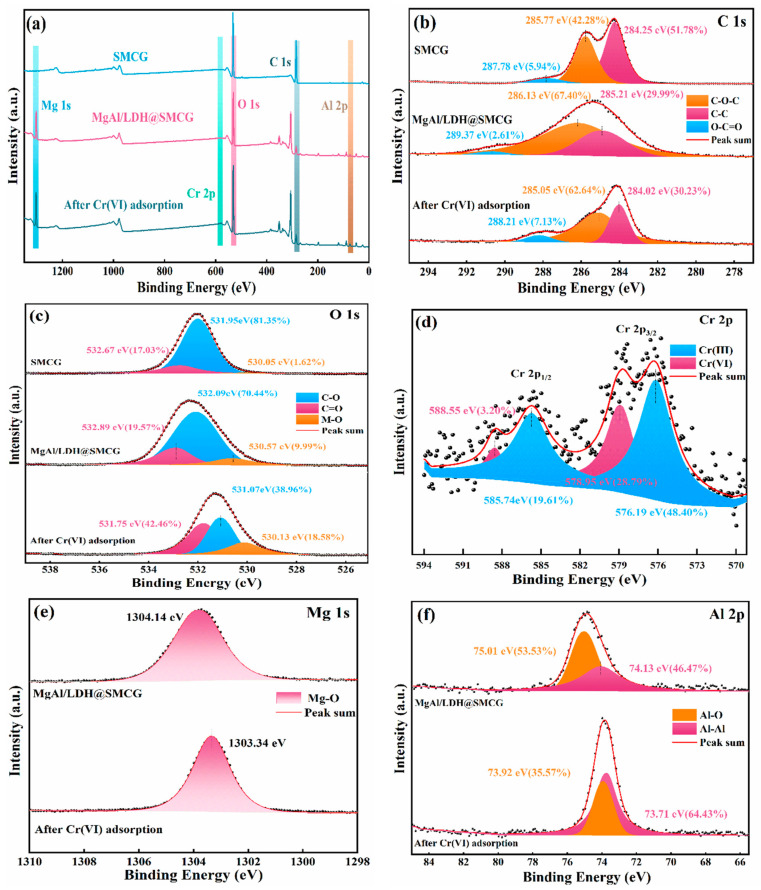
(**a**) XPS survey spectra of SMCG and MgAl/LDH@SMCG before and after Cr(VI) adsorption; high-resolution XPS spectra of (**b**) C 1s, (**c**) O 1s, (**d**) Cr 2p after Cr(VI) adsorption, and (**e**) Mg 1s and (**f**) Al 2p before and after Cr(VI) adsorption.

To further investigate the surface composition and chemical states, X-ray photoelectron spectroscopy (XPS) analysis was conducted on SMCG before and after modification, and on MgAl-LDH@SMCG following Cr(VI) adsorption. The results are presented in Figure 3. The full XPS spectra of the three materials (Figure 3a) show that the primary characteristic peaks of SMCG correspond to carbon and oxygen. After loading with MgAl LDH, MgAl-LDH@SMCG exhibited distinct Mg 1s and Al 2p peaks, which further confirms the successful incorporation of MgAl LDH. After Cr(VI) adsorption, two new peaks appeared in the 570–590 eV range of the MgAl-LDH@SMCG spectrum, corresponding to Cr 2p characteristic peaks and confirming the successful adsorption of Cr(VI) onto the material surface [36]. Figure 3b shows the deconvoluted C 1s spectrum, which reveals three distinct peaks corresponding to C–O–C, C=O, and C–C bonds. After adsorption, these peaks exhibited slight shifts, further confirming the complexation reaction between the adsorbent and Cr(VI) during the adsorption process [5].

Figure 3c presents the deconvoluted O 1s spectrum, where the characteristic peaks at 532.89 eV, 532.09 eV, and 530.57 eV are attributed to C=O, C–O, and M–O bonds, respectively [13]. Compared to pristine SMCG, the relative content of M–O bonds in MgAl-LDH@SMCG increased from 1.62% to 9.99%, which is attributed to the loading of MgAl LDHs that enhanced the overall M–O bond content (e.g., Mg–O and Al–O bonds). Following Cr(VI) adsorption, the relative content of M–O bonds increased significantly from 9.99% to 18.58%. This increase is due to the adsorption of a significant number of Cr(VI) ions on the material surface, leading to a marked rise in the Cr–O bond content. This observation aligns with the characteristic peak changes in the infrared spectra of MgAl-LDH@SMCG after Cr(VI) adsorption. In the Cr 2p region (Figure 3d), Cr(VI) adsorption is evidenced by two characteristic peaks corresponding to the Cr 2p1/2 and Cr 2p3/2 orbitals. The characteristic peaks at higher binding energies of 588.55 eV and 578.95 eV are attributed to Cr(VI), whereas the peaks at lower binding energies of 585.74 eV and 576.19 eV correspond to Cr(III) [13,37]. This indicates that during the adsorption process, a portion of Cr(VI) is reduced to Cr(III), primarily due to the role of C–O functional groups as electron donors [36,38]. This phenomenon is consistent with the significant decrease in the C–O bond content observed in MgAl-LDH@SMCG after chromium ion adsorption (Figure 3c). Furthermore, the presence of Cr(III) confirms that highly toxic Cr(VI) is effectively reduced to less toxic Cr(III) and immobilized on the adsorbent surface, enhancing the removal efficiency of Cr(VI) [7,39]. Figure 3e,f show the deconvoluted spectra of Al 2p and Mg 1s, respectively. After adsorption, the binding energies of Mg–O and Al–O shift to lower values, indicating their active involvement in the adsorption process [22]. The relative content of Al–O decreases, likely due to the activation of Al(III) within Al–O bonds under acidic conditions. Given the similar ionic radii of Al(III) and Cr(III), a portion of Al(III) ions may be substituted by Cr (III) ions [40]. This process alters the Al–O bond content and shifts the binding energy of the Al 2p peak.

### 2.4. TG Analysis

**Figure 4:**
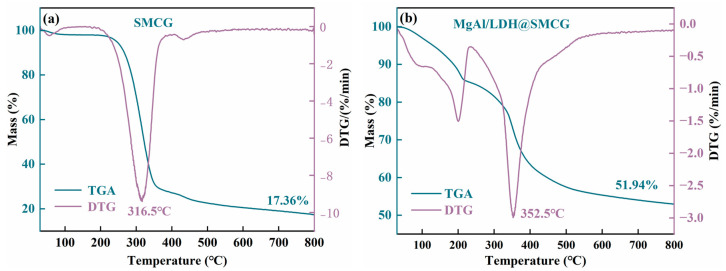
TG and DTG curves of the original SMCG and MgAl/LDH@SMCG: (**a**) Original SMCG; (**b**) MgAl/LDH@SMCG.

Thermogravimetric analysis (TG) and differential thermogravimetric analysis (DTG) were employed to assess the thermal stability of synthesized SMCG and MgAl-LDH@SMCG, with the corresponding curves shown in Figure 4. Figure 4 illustrates that the mass loss of sphagnum moss gel, both before and after modification, can be divided into three stages. In the first stage, mass loss occurred between 26.9 °C and 210 °C, primarily due to dehydration, including the evaporation of surface-adsorbed water and interlayer water [41]. The original SMCG showed slight mass loss at approximately 50 °C, primarily due to the evaporation of surface-adsorbed water, with a mass loss rate of 7.6%. After loading, the MgAl/LDH@SMCG exhibited a mass loss rate of 16.7%, attributed to both the evaporation of surface-adsorbed water and the release of interlayer water from MgAl/LDH domains, leading to significant mass loss at 198.3 °C. The second stage of mass loss occurred between 210 °C and 500 °C, representing the most significant mass loss and serving as a critical indicator of the material’s thermal stability. This stage includes the decomposition of the cellulose gel structure, the release of interlayer anions, and the final breakdown of the material’s structure [28,42]. Compared to the original SMCG, MgAl/LDH@SMCG showed a smoother weight loss curve, with the peak mass loss temperature (T_dm_) rising from 316.5 °C to 352.5 °C, indicating significantly improved thermal stability. The improved thermal stability is primarily due to hydrogen bonding between hydroxyl groups in the MgAl/LDH structure and functional groups on the SMCG surface. These interactions enhanced chain ordering and compactness, restricting molecular motion and thus improving thermal stability [43]. The third stage, observed between 500 °C and 800 °C, is mainly due to the combustion of residual materials [44]. At 800 °C, MgAl/LDH@SMCG exhibited a residue (M_800_) of 51.94%, significantly higher than the original SMCG, due to the decomposition of MgAl/LDH into magnesium and aluminum oxides.

### 2.5. Adsorption Performance Analysis

**Figure 5:**
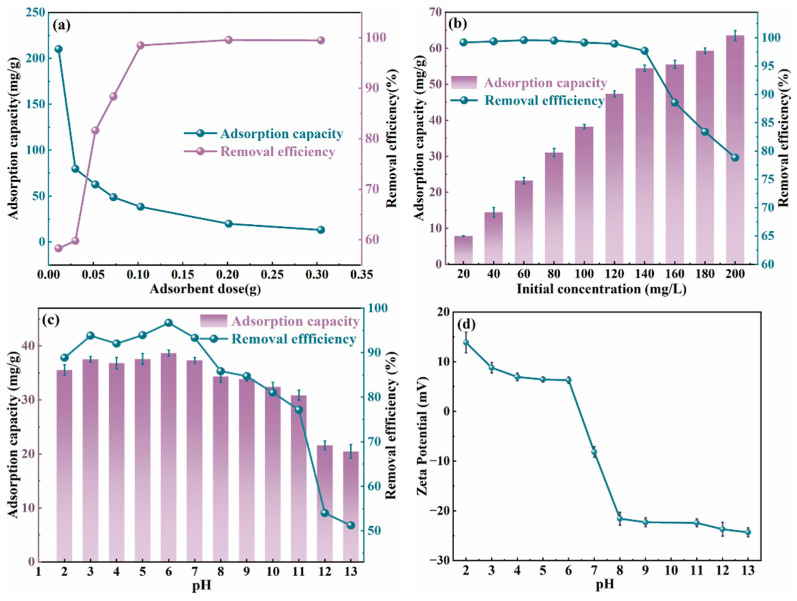
(**a**) Removal efficiency and adsorption capacity of Cr(VI) by MgAl/LDH@SMCG at different dosages. (**b**) Effect of initial Cr(VI) concentration on adsorption. (**c**) Cr(VI) adsorption behavior of MgAl/LDH@SMCG and (**d**) its zeta potential at various initial pH values.

The adsorbent dosage is a key determinant of the material’s adsorption performance. The effect of adsorbent dosage on adsorption performance was investigated under conditions of a 40 mL solution containing 100 mg/L Cr(VI) ion, pH 6, a contact time of 60 min, and a temperature of 303 K, with the results presented in Figure 5a. Figure 5a shows that increasing the adsorbent dosage from 0.01 g to 0.30 g raised the removal efficiency from 58.28% to 98.46%, while the adsorption capacity decreased from 210 mg/g to 13.06 mg/g. This occurs because higher adsorbent dosages provide more active sites for Cr(VI) ions, accelerating their binding and significantly improving removal efficiency. However, excessive adsorbent addition may reduce adsorption performance. At lower dosages, the surface adsorption groups of MgAl/LDH@SMCG fully interact with Cr(VI), effectively occupying active sites and increasing the adsorption capacity per unit mass. With increasing adsorbent dosage, the contact between MgAl/LDH@SMCG and Cr(VI) per unit mass decreases, leaving the active sites unsaturated. Excessive adsorbent dosage may cause over-suspension, resulting in aggregate formation, reduced specific surface area, hindered contact with Cr(VI), and decreased adsorption site utilization, ultimately impairing overall adsorption performance [5,45,46]. From a practical perspective, optimizing the adsorbent dosage to achieve a balance between high removal efficiency and adsorption capacity can effectively minimize costs. Experimental findings indicate that the optimal dosage of MgAl/LDH@SMCG is 0.10 g.

The choice of initial concentration plays a critical role in the adsorption process of the material. Figure 5b illustrates the changes in adsorption capacity and removal efficiency of MgAl/LDH@SMCG at different initial Cr(VI) concentrations (20–200 mg/L). The experiments were conducted under conditions of pH 6, an adsorbent dosage of 0.1 g, a contact time of 60 min, and a temperature of 303 K. The results show that the Cr(VI) concentration has a significant impact on adsorption efficiency. Within the range of 20–200 mg/L, the adsorption capacity of Cr(VI) increased from 7.85 mg/g to 63.57 mg/g. However, the removal efficiency showed an inverse relationship with the initial Cr(VI) concentration, decreasing from 99.55% to 78.81%. This phenomenon is mainly due to the higher initial Cr(VI) concentration, which strengthens the adsorption driving force, promotes ion diffusion toward the adsorbent, and intensifies collision effects between the adsorbate and adsorbent, leading to increased adsorption capacity [2]. However, higher Cr(VI) concentrations in the solution notably reduce the removal efficiency. This reduction is primarily caused by the limited active adsorption sites on the MgAl/LDH@SMCG surface, which lower the affinity for transferring Cr(VI) from the suspension to the adsorbent. Consequently, the adsorption performance reaches saturation, limiting the further uptake of Cr(VI) and reducing the removal efficiency. In summary, these results confirm that the initial Cr(VI) concentration is a critical determinant of the adsorption performance of MgAl/LDH@SMCG.

The pH of the solution is a key factor influencing the adsorption efficiency of the adsorbent during the adsorption process. In this study, pH-dependent adsorption experiments were conducted using an initial Cr(VI) concentration of 100 mg/L to evaluate the adsorption behavior of MgAl/LDH@SMCG under varying acidic and alkaline conditions. Changes in pH notably affect the surface charge distribution of the supported layered double hydroxide (LDH) gel and the chemical speciation of Cr(VI), significantly impacting the adsorption performance of the adsorbent. Chromium (Cr) presents different ionic forms under varying pH conditions, with trivalent chromium (Cr(III)) and hexavalent chromium (Cr(VI)) being the most prevalent species [47].

As shown in Figure 5c,d, MgAl-LDH@SMCG exhibits a positive charge over a broad pH range (pH = 2.0–6.0), with chromium removal efficiency increasing from 87.4% to 97.08%. Correspondingly, the adsorption capacity also increased from 35.54 mg/g to 38.67 mg/g within this pH range. This improvement is primarily due to the reduction of Cr(VI) ions to Cr(III) ions, such as Cr^3+^ and Cr(OH)^2+^, under acidic conditions (low pH), where these ions exist in positively charged forms [48]. Positively charged Cr(III) ions undergo electrostatic repulsion with the positively charged surface of MgAl-LDH@SMCG, reducing the removal efficiency of Cr(VI). At low pH, Cr(VI) ions primarily exist as HCrO_4_^−^, exhibiting weaker electrostatic attraction to the MgAl-LDH@SMCG surface compared to other species, such as Cr_2_O_7_^2−^ and CrO_4_^2−^ [40]. Additionally, at low pH, the dissolution of metal hydroxides can cause the structural collapse of LDH, which is another reason for the reduced adsorption efficiency [40,49]. As pH increases, Cr(VI) ions predominantly exist as HCrO_4_^−^ and Cr_2_O_7_^2−^ and their enhanced negative charge promotes electrostatic adsorption onto the positively charged MgAl-LDH@SMCG, thereby improving adsorption performance [47]. When pH exceeds 6, Cr(VI) ions in the waste solution primarily exist as CrO_4_^2−^ . At this stage, competitive adsorption of hydroxide ions and electrostatic repulsion between the adsorbent and chromium anions significantly reduce adsorption capacity. It is evident that the pH value primarily enhances the adsorption efficiency of Cr(VI) by modulating the active binding sites on the adsorbent surface. Therefore, selecting an appropriate pH value is critical for a detailed investigation of Cr(VI) adsorption performance.

### 2.6. Adsorption Kinetics

**Figure 6:**
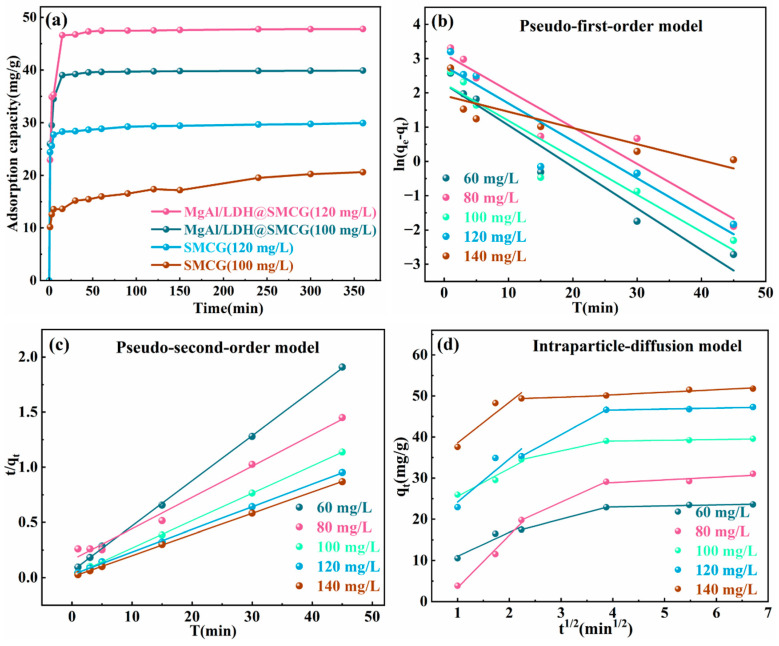
(**a**) Effect of contact time on the adsorption performance of SMCG and MgAl/LDH@SMCG; adsorption kinetics of MgAl/LDH@SMCG fitted to (**b**) the pseudo-first-order model, (**c**) the pseudo-second-order model, and (**d**) the intraparticle diffusion model.

Contact time is a crucial factor influencing the efficiency of Cr(VI) ion removal during the adsorption process. The adsorption capacity of the material was measured under conditions of 40 mL Cr(VI) solutions with concentrations of 60–140 mg/L, pH 6, an adsorbent dose of 0.1 g, and a temperature of 303 K, at varying contact times. The adsorption kinetics of sphagnum moss gel (SMCG) before and after modification were analyzed over time, and the results are shown in Figure 6. Figure 6 illustrates that the adsorption capacities of SMCG and MgAl/LDH@SMCG increase with contact time, reaching equilibrium within 60 min. This behavior is primarily attributed to the synergistic effect of the material’s porous structure and abundant surface-active sites. Notably, the modified composite exhibited a Cr(VI) adsorption capacity of 47.5 mg/g and with a high removal efficiency of 97.08%, significantly surpassing both the unmodified SMCG and many reported bio-based adsorbents [33,35,37,50]. This improvement is attributed to the optimized gel surface structure after MgAl/LDH loading, which increases the specific surface area and the number of adsorption-active sites, thereby significantly enhancing adsorption performance. With extended adsorption time, available adsorption sites are gradually occupied, and the migration rate of Cr(VI) to the pore surfaces decreases. Some active adsorption sites may become blocked, reducing the adsorption rate and eventually causing the adsorption capacity to stabilize [4].

The study of adsorption kinetics is crucial for understanding the interactions between adsorbents and adsorbates, as well as for determining the mechanisms underlying the adsorption process. To investigate the adsorption kinetics mechanism of MgAl/LDH@SMCG for Cr(VI), pseudo-first-order, pseudo-second-order, and intraparticle diffusion models were employed to fit the experimental data. The fitting equations are shown as Equations (1)–(3) [7,17]:

(1) $$\ln\left(q_{e}-q_{t}\right)=\ln q_{e}-K_{1}t$$

(2) $$\frac{t}{q_{t}}=\frac{1}{K_{2}q_{e}^{2}}+\frac{t}{q_{e}}$$

(3) $$q_{t}=K_{i}t^{\frac{1}{2}}+C$$

In the equations, $q_{e}$ (mg/g) represents the equilibrium adsorption capacity of Cr(VI), while $q_{t}$ (mg/g) denotes the adsorption capacity of Cr(VI) at time *t* (min). *K*_1_ (min^−1^) and *K*_2_ (g·mg^−1^·min^−1^) are the pseudo-first-order and pseudo-second-order rate constants, respectively. *K_i_* (mg·g^−1^·min^−1/2^) represents the intraparticle diffusion constant, and *C* (mg·g^−1^) is associated with the boundary layer thickness.

The fitting curves for the pseudo-first-order, pseudo-second-order, and intraparticle diffusion models are illustrated in Figure 6b–d, with the corresponding fitting parameters provided in Table 1. The pseudo-second-order kinetic model demonstrates higher correlation coefficients and a calculated equilibrium adsorption capacity closer to the experimental values compared to the pseudo-first-order model, indicating its greater suitability for describing the material’s adsorption behavior. This finding further indicates that Cr(VI) removal is primarily governed by chemisorption, which involves electron transfer [51]. Figure 6d illustrates the intraparticle diffusion model’s fitting curve, displaying a three-stage linear pattern. This suggests that the adsorption of Cr(VI) by MgAl/LDH@SMCG occurs in three distinct stages, governed by multiple mechanisms [27,37]. During the first stage, the steep slope is primarily attributed to surface diffusion, where a large number of Cr(VI) diffuse to the adsorbent surface and interact with abundant adsorption sites, facilitating rapid adsorption reactions. In the second stage, the slope decreases as effective surface adsorption sites are gradually occupied, leading to a reduced adsorption rate, which is primarily governed by intraparticle diffusion. Compared to the first stage, this phase requires a longer adsorption time, and the rate-controlling step may be attributed to either intraparticle or pore diffusion [52]. In the third stage, the slope approaches zero, indicating that the adsorption process enters the slowest equilibrium phase. At this stage, surface adsorption sites are nearly saturated, and the material’s adsorption capacity reaches its maximum. This phase likely involves the migration of Cr(VI) between adsorbent particles, enabling them to occupy additional adsorption sites [39,52]. The fitting curve does not pass through the origin, indicating that intraparticle diffusion is not the sole factor controlling the adsorption process. Boundary layer diffusion and external mass transfer may also contribute significantly [53].

### 2.7. Adsorption Isotherms

Adsorption isotherms describe the dynamic equilibrium between adsorbates and adsorbents at varying concentrations under specific temperature conditions, providing an important basis for studying adsorption behavior [39]. Adsorption equilibrium experiments were conducted using Cr(VI) solutions with concentrations ranging from 80 to 200 mg/L. The experimental conditions included a solution pH of 6, an adsorbent dosage of 0.1 g, a contact time of 5 h, and adsorption temperatures of 303 K, 313 K, 323 K, and 333 K. The adsorption capacity of the material at various temperatures was measured as a function of concentration. Adsorption isotherm models were applied to fit the experimental data.

**Figure 7:**
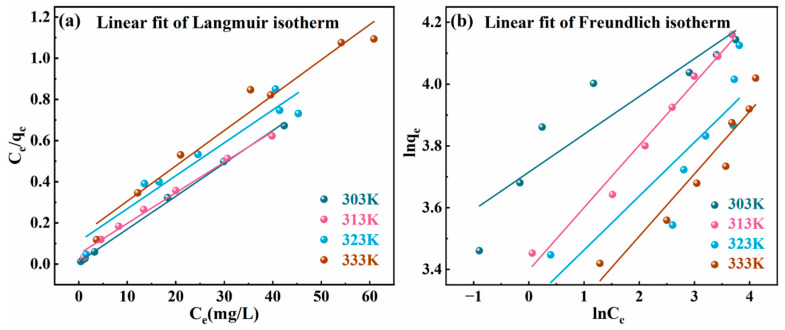
Adsorption isotherm model for removal of MgAl/LDH@SMCG adsorbents: (**a**) Langmuir adsorption isotherm model; (**b**) Freundlich adsorption isotherm model.

The adsorption mechanism of MgAl/LDH@SMCG for Cr(VI) ions was investigated, and the fitting results are shown in Figure 7 and Table 2. The Langmuir and Freundlich isotherm models are widely used to describe adsorption behavior. The Langmuir model describes monolayer chemical adsorption on a homogeneous surface. It assumes uniformly distributed adsorption sites, with no interactions between sites, and exhibiting similar adsorption properties. The Freundlich model is an empirical equation that describes multilayer adsorption, considers interactions between adsorption sites, and reveals the heterogeneous energy distribution. The corresponding equations are shown as Equations (4) and (5) [23,37]:

(4) $$\frac{C_{e}}{q_{e}}=\frac{C_{e}}{q_{m}}+\frac{1}{K_{L}q_{m}}$$

(5) $$\ln q_{e}=\ln K_{F}+\frac{1}{n}\ln c_{e}$$

In the equations, $q_{e}(mg\cdot g^{-1})$ indicates the equilibrium adsorption capacity, $q_{m}(mg\cdot g^{-1})$ represents the saturated adsorption capacity, and $C_{e}$(mg/L) denotes the Cr(VI) concentration in solution at adsorption equilibrium. $K_{L}$ is the Langmuir constant, which is associated with adsorption free energy and the affinity of adsorption sites. The Freundlich constants $K_{F}$ and n indicate the system’s adsorption capacity and adsorption intensity, respectively.

The fitting results in Figure 7 and Table 2 show that the Langmuir model has a significantly higher correlation coefficient ($R^{2}$) than the Freundlich model, suggesting that the Langmuir model provides a more accurate fit and better describes the adsorption behavior of Cr(VI) on the adsorbent. Cr(VI) is primarily adsorbed at specific active sites on the adsorbent surface, exhibiting high adsorption affinity under high-concentration conditions. The adsorption mechanism is primarily monolayer adsorption [33]. The experimentally measured equilibrium adsorption capacity closely matches the theoretical maximum predicted by the Langmuir model, confirming that the adsorption of Cr(VI) by MgAl/LDH@SMCG follows the characteristics of a monolayer adsorption model.

### 2.8. Thermodynamics Investigation

Thermodynamic analysis plays a pivotal role in assessing the feasibility and spontaneity of adsorption processes. Analyzing changes in Gibbs free energy, enthalpy, and entropy reveals the energy variation patterns of the adsorption process and elucidates the interaction mechanisms between adsorption sites and Cr(VI). The changes in Gibbs free energy ($∆G^{0}$), enthalpy ($∆H^{0}$), and entropy ($∆S^{0}$) during the adsorption process can be calculated using the following equations [12]:

(6) $$K_{c}=\frac{q_{e}}{C_{e}}$$

(7) $$\text{∆}G^{0}=-RT\ln K_{c}$$

(8) $$\ln K_{c}=\frac{∆S^{0}}{R}-\frac{∆H^{0}}{RT}$$

In the equations, $K_{c}$ represents the thermodynamic equilibrium constant, while $q_{e}(mg\cdot g^{-1})$ and $C_{e}\left(mg\cdot L^{-1}\right)$ denote the equilibrium adsorption capacity and equilibrium concentration of Cr(VI), respectively. The ideal gas constant $R(J\cdot {mol}^{-1}\cdot K^{-1})$ is 8.314, and T (K) denotes the absolute temperature.

**Figure 8:**
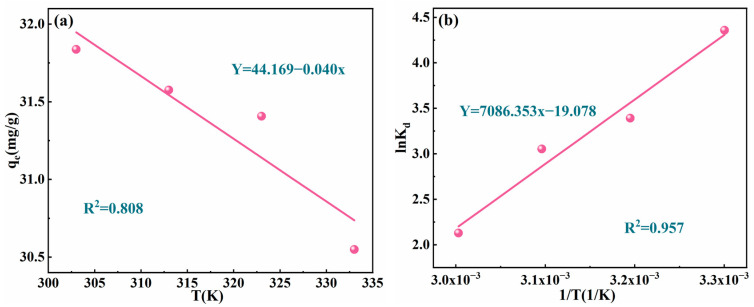
Thermodynamic fitting of MgAl/LDH@SMCG: (**a**) adsorption capacity as a function of temperature; (**b**) linear fitting curve of the reciprocal temperature.

To investigate the thermodynamic behavior of the material’s adsorption, equilibrium adsorption experiments were conducted under the following conditions: pH 6, an initial concentration of 100 mg/L, an adsorbent dosage of 0.1 g, a contact time of 5 h, and temperatures of 303 K, 313 K, 323 K, and 333 K. Adsorption capacities at different temperatures were measured to analyze the temperature-dependent trends of MgAl/LDH@SMCG and to investigate its thermodynamic characteristics. The fitting results and thermodynamic parameters are shown in Figure 8 and Table 3. Figure 8a illustrates that the adsorption capacity of MgAl/LDH@SMCG for Cr(VI) ions decreases as the temperature increases, indicating that the adsorption process is exothermic [36]. The values of ΔH^0^ and ΔS^0^ can be determined from the slope and intercept of the $\ln K_{c}$ versus $\frac{1}{T}$ curve (Figure 8b). The ΔH^0^ value of −58.916 kJ/mol suggests that the adsorption process is predominantly driven by chemical adsorption [54]. The ΔS^0^ value is negative, indicating that the adsorption results in a decrease in system entropy. During the adsorption process, the negative ΔG^0^ value confirms that the adsorption reaction occurs spontaneously and is feasible without requiring specific external conditions [53]. As temperature increases, the $K_{c}$ value decreases, suggesting reduced affinity of MgAl/LDH@SMCG for Cr(VI) ions. Concurrently, the ΔG^0^ value increases, indicating that lower temperatures favor Cr(VI) adsorption.

### 2.9. Adsorption Mechanism

**Figure 9:**
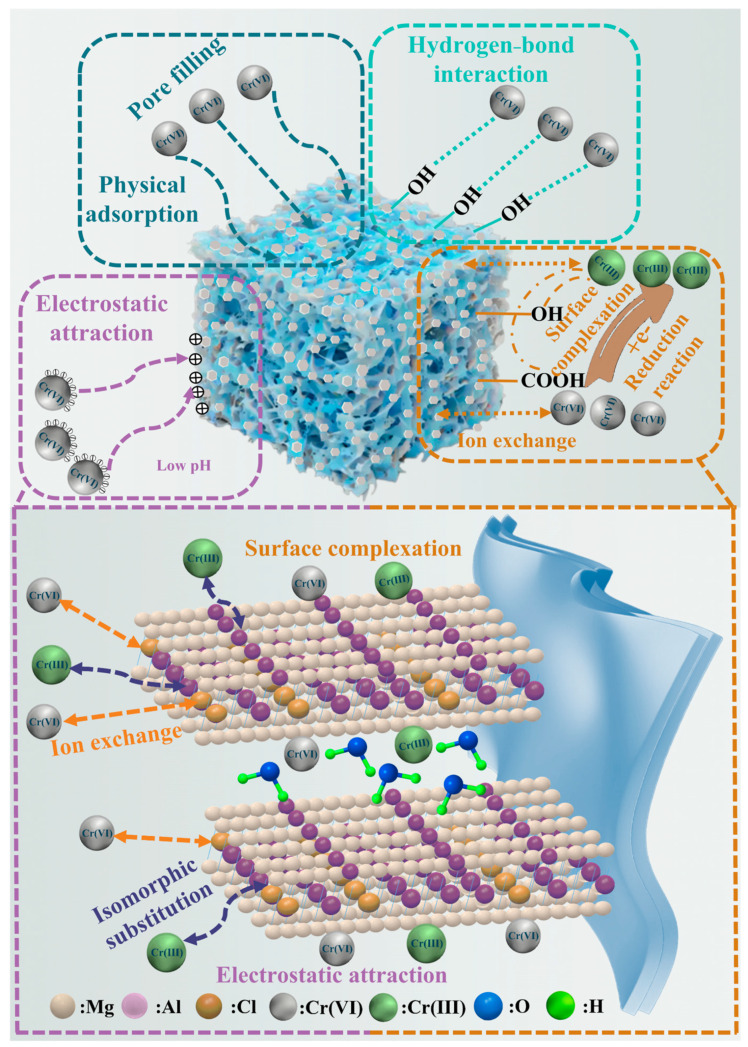
Schematic diagram of the adsorption−reduction mechanism of Cr ion on MgAl/LDH@SMCG.

The adsorption process primarily results from interactions between active sites on the adsorbent surface and pollutant molecules. During Cr(VI) adsorption in water, MgAl/LDH@SMCG demonstrates multiple synergistic mechanisms, as illustrated in Figure 9. The SMCG surface is smooth and flat, with cellulose crosslinking forming a highly ordered mesh-like cavity that serves as the foundation for efficient adsorption. After loading with magnesium–aluminum layered double hydroxides (MgAl/LDH), the surface roughness of MgAl/LDH@SMCG significantly increases while maintaining its original porous framework and forming a multilevel fibrous channel structure. This structure facilitates the rapid migration, diffusion, and localization of Cr(VI) in aqueous solutions and also acts as a carrier for the uniform dispersion of MgAl/LDH, further enhancing active site exposure. This pore diffusion mechanism enables Cr(VI) to rapidly penetrate the material’s interior, access more active adsorption sites, and significantly enhance overall adsorption efficiency [27].

In MgAl/LDH@SMCG, the abundant hydroxyl groups (–OH) on the MgAl/LDH surface and the C-OH groups in the SMCG gel framework play a crucial role in the adsorption process. Under acidic conditions, hydroxyl groups undergo protonation, enhancing their polarization interaction with oxygen atoms in Cr(VI) anions (e.g., HCrO_4_^−^ and Cr_2_O_7_^2−^). This process leads to the formation of stable hydrogen bonds, facilitating the adsorption of Cr(VI) onto the MgAl/LDH@SMCG surface [19,40,55]. The deconvoluted O 1s spectrum from XPS further confirms that, following Cr(VI) adsorption, the C–O bond shifts to lower binding energies, suggesting strong electronic interactions between oxygen atoms and hydroxyl groups.

Electrostatic adsorption plays a crucial role in Cr(VI) removal, as the surface charge properties of the adsorbent directly determine its interaction with charged pollutants. After loading with MgAl/LDH, the SMCG gel surface becomes positively charged. Under acidic conditions, further protonation enhances electrostatic attraction with Cr(VI) anions (e.g., HCrO_4_^−^ and Cr_2_O_7_^2−^) in solution, leading to efficient Cr(VI) removal [1,3]. In an alkaline environment, the functional groups on the MgAl/LDH@SMCG surface deprotonate, causing the surface to become negatively charged. Consequently, electrostatic repulsion occurs between the surface and Cr(VI) anions (e.g., CrO_4_^2−^) in solution. Meanwhile, OH^−^ ions compete with Cr(VI) for adsorption sites, leading to a reduction in adsorption efficiency [6,56]. This adsorption mechanism is consistent with experimental findings regarding the effect of pH on adsorption performance.

The MgAl/LDH layered double hydroxide (LDH) on the surface of MgAl/LDH@SMCG offers an efficient platform for ion exchange during adsorption due to its unique layered structure and the exchangeable nature of its interlayer anions. During adsorption, the high specific surface area of the MgAl/LDH layers promotes extensive contact with Cr(VI) anions (e.g., HCrO_4_^−^, Cr_2_O_7_^2−^) in the solution. These anions subsequently migrate into the LDH interlayers through ion exchange, replacing the original interlayer anions. Simultaneously, Cr(VI) anions are strongly bound to the positively charged LDH layers through electrostatic interactions, ensuring stable adsorption [51]. Furthermore, during Cr(VI) adsorption, the reductive C-OH groups on the MgAl/LDH@SMCG surface oxidize to C=O, as indicated by changes in the intensity of C–O and C=O peaks in the XPS spectrum. Simultaneously, these groups act as electron donors, reducing Cr(VI) to Cr(III) [13]. The reduced Cr(III) ions participate in ion exchange with Al^3+^ ions on the adsorbent surface, promoting their incorporation and improving adsorption capacity [40,57]. Additionally, Cr(III) can also be adsorbed through surface complexation with carboxyl (–COOH) and hydroxyl (–OH) groups on the MgAl/LDH@SMCG surface, further stabilizing the adsorption structure [58]. This multimechanism adsorption model provides theoretical insights and practical guidance for adsorbent design and water treatment applications. Furthermore, it provides insights into the functional surface modification of future materials.

## 3. Experimental Methods

### 3.1. Materials

The gel adsorption material was prepared using raw sphagnum moss as the base material, sourced from a local supplier in Guizhou, China. Sodium hydroxide (NaOH) and sodium chlorite (NaClO_2_), used for the pretreatment and cellulose extraction of sphagnum moss, were obtained from Tianjin Kemiou Chemical Reagent Co., Ltd. (Tianjin, China). Polyvinyl alcohol (PVA), used in the preparation of sphagnum-based cellulose aerogels, was obtained from Shandong Xuchen Chemical Technology Co., Ltd. (Xuchen, China). The modification reagents, aluminum chloride (AlCl_3_·6H_2_O, analytical grade) and magnesium chloride (MgCl_2_·6H_2_O, analytical grade), were supplied by Chongqing Wansheng Chemical Co., Ltd. (Chongqing, China). Other reagents, such as potassium dichromate (K_2_Cr_2_O_7_), acetic acid (CH_3_COOH), sulfuric acid (H_2_SO_4_), phosphoric acid (H_3_PO_4_), hydrochloric acid (HCl), and potassium bromide (KBr), were purchased from Shandong Hichem Chemical Group Co., Ltd. (Qingdao, China). All chemicals utilized in the experiments were of analytical grade and used without further purification.

### 3.2. Preparation of Gel Adsorption Materials

Ten grams of pulverized sphagnum moss was added to a beaker containing 400 mL of 1.6 mol/L NaOH solution and stirred with a magnetic stirrer in a water bath at 80 °C for 3 h. After cooling, the sample was washed to neutrality, filtered, and dried in an oven at 80 °C to obtain pretreated sphagnum moss. The pretreated sphagnum moss was added to 200 mL of 0.89 mol/L NaClO_2_ solution and stirred at 75 °C for 3 h. The sample was then washed to neutrality, vacuum filtered, and reacted in 1 mol/L NaOH solution at 75 °C for 6 h. Finally, the sample was washed, vacuum filtered, and dried to yield sphagnum moss cellulose. The extracted sphagnum moss cellulose was mixed with 200 mL of 25 g/L PVA solution, ultrasonically dispersed for 30 min, and uniformly reacted at 95 °C for 1 h. The resulting mixture was poured into a mold, frozen at −30 °C, and freeze-dried for 72 h to form sphagnum moss cellulose gel (SMCG).

**Figure 10:**
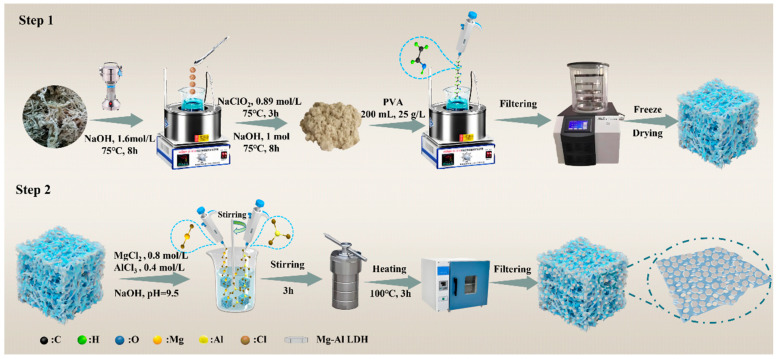
Schematic illustration of the synthesis process of MgAl/LDH@SMCG with high Cr(VI) adsorption efficiency.

Two grams of cellulose gel was mixed with a solution containing 60 mL of 0.8 mol/L MgCl_2_ and 60 mL of 0.4 mol/L AlCl_3_, followed by pH adjustment to 9.5 using 5 mol/L NaOH. The mixture was stirred at room temperature using a magnetic stirrer (IKA, Staufen, Germany) for 3 h, and then transferred to a reaction vessel with a polytetrafluoroethylene lining, treated at 100 °C in a muffle furnace for 3 h. After treatment, the product was washed with deionized water to neutrality, vacuum filtered, and freeze-dried to yield Mg-Al-loaded sphagnum moss cellulose gel (MgAl/LDH@SMCG). Compared with traditional high-temperature calcination or chemical vapor deposition techniques, this method avoids extreme reaction conditions and significantly reduces energy consumption. Moreover, the low-temperature synthesis and freeze-drying processes effectively preserve the porous three-dimensional structure of the gel, ensuring its adsorption performance and structural integrity for potential large-scale applications. The detailed synthesis process is illustrated in Figure 10.

### 3.3. Characterization

The surface morphology of sphagnum moss cellulose gel (SMCG) before and after modification was examined using field-emission scanning electron microscopy (FE-SEM; Gemini 300, Zeiss, Oberkochen, Germany) operated at an accelerating voltage of 5 kV to obtain high-resolution images. Prior to analysis, the samples were sputter-coated with a thin layer of gold to improve surface conductivity. Elemental composition was analyzed using energy-dispersive X-ray spectroscopy (EDS; X-MaxN 80, Oxford Instruments, Abingdon, UK) integrated with the SEM, operated at an accelerating voltage of 15 kV to ensure optimal X-ray excitation and detection. Fourier transform infrared spectroscopy (FT-IR; Tensor 27, Bruker, Karlsruhe, Germany) was employed to analyze the surface chemical functional groups of SMCG and MgAl/LDH@SMCG cellulose gels. The samples were dried at 150 °C for 2 h and analyzed using the KBr pellet method across the wavenumber range of 400–4000 cm^−1^ to identify chemical functional groups. Thermogravimetric analysis (TGA) was conducted using a Netzsch TG209F1 analyzer (Netzsch, Sable, Germany) to evaluate the thermal stability of the original SMCG and MgAl/LDH@SMCG. Approximately 5.59 mg of each sample was loaded into an alumina crucible and heated from ambient temperature to 900 °C under a nitrogen atmosphere at a heating rate of 10 °C/min and a nitrogen flow rate of 20 mL/min. Zeta potential measurements of MgAl/LDH@SMCG under different pH conditions were performed using a Zetasizer (Nano ZS90, Malvern Instruments, Malvern, UK) equipped with a 633 nm He–Ne laser and a backscatter detector (173° detection angle). All measurements were carried out at 25 ± 0.1 °C in disposable folded capillary cells. Prior to analysis, samples were ultrasonicated for 10 min and equilibrated at the measurement temperature. The Z-average (Z-AVE) particle size was determined by dynamic light scattering (DLS), and zeta potential values were calculated based on electrophoretic mobility using the Smoluchowski approximation. Each reported value represents the average of three independent measurements. X-ray photoelectron spectroscopy (XPS; K-Alpha, Thermo Scientific, Waltham, MA, USA) was employed to investigate the surface elemental composition and chemical states of SMCG before and after surface modification. Measurements were conducted using a monochromatic Al Kα X-ray source (hv = 1486.6 eV) operated at 12 kV and 6 mA. Spectra were acquired over a scan area of 400 μm × 400 μm with a photoelectron take-off angle of 90°. Survey spectra (0–1350 eV) were collected at a pass energy of 150 eV with a step size of 1.0 eV, while high-resolution spectra were recorded at a pass energy of 50 eV and a step size of 0.1 eV. All binding energies were calibrated against the adventitious carbon C 1s peak at 284.8 eV to correct for charging effects. The base pressure in the analytical chamber was maintained below 1.0 × 10^−9^ mbar throughout the analysis.

### 3.4. Adsorption Experiments

An appropriate quantity of potassium dichromate (K_2_Cr_2_O_7_, > 99%, Sigma-Aldrich, St. Louis, MO, USA) was dissolved in deionized water to prepare a 200 mg/L Cr(VI) standard solution. Batch adsorption experiments were performed to evaluate the influence of adsorption time (0–360 min), pH (2–13), initial concentration (80–200 mg/L), adsorption temperature (303–333 K), and adsorbent dosage (0–0.30 mg/L) on adsorption performance. The Cr(VI) concentration and pH values used in this study were selected as representative parameters to investigate the adsorption behavior and mechanism of the MgAl/LDH@SMCG composite. Specifically, pH 6 was chosen due to its significance in Cr(VI) speciation and its frequent use in adsorption studies, while the concentration range of 20–200 mg/L covers both moderate and high contamination levels commonly reported in the literature. These conditions allow for mechanistic insights under practically relevant scenarios. For all experiments except those testing adsorbent dosage, 0.10 g of MgAl/LDH@SMCG adsorbent was added to 40 mL of Cr(VI) solution after pH adjustment. The mixture was stirred at 293 K and 220 r/min for 60 min until adsorption equilibrium was reached. To minimize random errors and enhance data reliability, equilibrium adsorption experiments were conducted in triplicate across multiple independently synthesized batches of the composite. Each experimental set included three parallel trials using independently prepared samples to ensure reproducibility.

Following adsorption equilibrium, the adsorbent and Cr(VI) solution were separated by high-speed centrifugation. The coloring reagent was prepared by dissolving 0.5 g of diphenylcarbazide (C_13_H_14_N_4_O) and 4 g of phthalic anhydride (C_8_H_4_O_3_) in anhydrous ethanol and diluting to 100 mL. Five milliliters of the coloring reagent, combined with 1 mL of phosphoric acid (H_3_PO_4_, 6.78 mol/L) and 1 mL of sulfuric acid (H_2_SO_4_, 9.20 mol/L), was added to 5 mL of the centrifuged supernatant and then diluted to 25 mL. The concentration of residual Cr(VI) in the supernatant was measured using spectrophotometry. The supernatant absorbance was measured at 540 nm using a P4 UV-vis spectrophotometer (MAPADA, Shanghai, China). To ensure result accuracy and reproducibility, each sample was tested three times, and the average value was calculated to determine the adsorption capacity and removal efficiency of the Cr(VI) ion solution using Equations (9) and (10):

(9) $$\mathrm{Adsorption}\;\mathrm{capacity}\text{:}\;q_{e}=\frac{{(C}_{0}-C_{e})V}{m}$$

(10) $$\mathrm{Removal}\;\mathrm{efficiency}\text{:}\;R=\frac{C_{0}-C_{e}}{C_{0}}\times 100\%$$

$C_{e}\;$ and $C_{0}$ represent the residual and initial Cr(VI) concentrations in the solution (mg/L), respectively; m denotes the mass of the MgAl/LDH@SMCG adsorbent (g), and *V* refers to the initial volume of the solution (L).

## 4. Conclusions

This study developed a Mg/Al layered double hydroxide-modified sphagnum moss cellulose gel composite (MgAl/LDH@SMCG) for efficient Cr(VI) removal from aqueous solutions. The material exhibited a highly porous structure, abundant active sites, and good thermal stability, contributing to its high adsorption capacity. Batch experiments showed a maximum Cr(VI) removal efficiency of 97.08% under optimal conditions (pH 6, 60 min). The adsorption followed pseudo-second-order kinetics and Langmuir isotherm behavior, suggesting a chemisorption-dominated monolayer mechanism. Thermodynamic and mechanistic analyses revealed that Cr(VI) removal involved electrostatic attraction, hydrogen bonding, ion exchange, surface complexation, and partial reduction to Cr(III). Despite its promising performance, the material’s long-term stability, regeneration capacity, and performance in complex water matrices require further investigation. Future studies should also explore scale-up potential and environmental impacts to fully assess its practical applicability.

## Figures and Tables

**Table 1:** Comparison of PFO and PSO kinetic model fitting for adsorption data of all samples.

	Parameters	Initial Concentration (mg/L)				
		60	80	100	120	140
	$q_{e}$, exp(mg·g^−1^)	23.6445	31.1871	39.6445	47.4609	52.8479
Pseudo-first- order model	K_1_ (min^−1^)	0.1211	0.1067	0.1080	0.1091	0.0472
	R^2^	0.9398	0.9101	0.8931	0.8704	0.7531
	$q_{e\;}$(mg·g^−1^)	9.6194	22.8902	9.6549	16.1710	6.8210
Pseudo-second-order model	K_2_ (g·mg^−1^·min^−1^)	0.0282	0.0050	0.0329	0.0163	0.0523
	R^2^	0.9995	0.9868	0.9998	0.9994	0.9999
	$q_{e\;}$(mg·g^−1^)	24.4559	35.3107	40.2576	48.7567	52.6367

**Table 2:** Adsorption isotherm model fitting parameters.

Adsorption Isotherm Models	Parameters	303 K	313 K	323 K	333 K
Langmuir	*q_m_*	62.6566	67.2495	62.5391	58.3090
	K_L_	1.4888	0.3035	0.1459	0.1275
	R_L_	0.0083	0.0396	0.0789	0.0893
	R^2^	0.9978	0.9913	0.9149	0.9626
Freundlich	K_F_	41.0767	29.9129	26.8056	22.2771
	1/*n*	0.1222	0.2019	0.1744	0.2020
	R^2^	0.8103	0.9788	0.6903	0.8951

**Table 3:** Adsorption thermodynamic parameters for Cr(VI) adsorption of MgAl/LDH@SMCG.

ΔH^0^ (kJ/mol)	ΔS^0^ (J/(mol·K)	ΔG^0^ (kJ/mol)			
−58.916	−158.615	303 K	313 K	323 K	333 K
		−10.980	−8.828	−8.198	−5.899

## Data Availability

Data are provided within the manuscript and available on request from Yu Wang (wangyugeu@126.com).
